# Emission Properties, Solubility, Thermodynamic Analysis and NMR Studies of Rare-Earth Complexes with Two Different Phosphine Oxides

**DOI:** 10.3390/ma3084080

**Published:** 2010-07-26

**Authors:** Hiroki Iwanaga

**Affiliations:** Corporate Research & Development Centre, Toshiba Corporation, 1 Komukai-Toshiba-cho, Saiwai-ku, Kawasaki 212-8582, Japan; E-Mail: hiroki.iwanaga@toshiba.co.jp; Tel.: +81-44-549-2359; Fax: +81-44-520-1255

**Keywords:** Eu(III) complex, Tb(III) complex, bis-phosphine oxide, solubility, emission intensity, LED

## Abstract

The paper proposes novel molecular designs for rare-earth complexes involving the introduction of two different phosphine oxide structures into one rare-earth ion. These designs are effective for improving solubility and emission intensity. Additionally, the complexes are indispensable for realizing high performances in LEDs and security media. The thermodynamic properties of Eu(III) complexes are correlated with the solubility. Correlations between coordination structures and emission intensity were explained by NMR analysis. The luminous flux of red LED devices with Eu(III) complexes is very high (20 mA, 870 m lumen). A new white LED has its largest spectra intensity in the red region and a human looks much more vividly under this light.

## 1. Introduction

Rare-earth complexes, notably Eu(III) complexes, have been extensively investigated as luminescent materials because of their potential for use in emission devices, security media, ornaments, and many other applications [1,2,3,4,5,6,7,8,9,10]. 

Rare-earth complexes have some notable features. In the first place, when they are dissolved, their emission spectra change little according to their concentration and the matrix polymer solvent. When luminescent materials are used in LED devices, their emission spectra must be adjusted to the suitable chromaticity coordinates and color temperature fitted to the application. These parameters are mainly determined by the balance of intensity, the maximum wavelength, and the half width of the emission spectra of both luminescent materials and LED chips. The emission spectra of many organic luminescent materials are shifted according to their concentration and the matrix polymer in which they are dissolved. Thus, adjusting the emission spectra of LED devices is very difficult. On the other hand, the emission spectra of rare-earth complexes shift little, due to the effects of having f-f transition, which is very advantageous for adjusting the emission spectra of LED devices. 

Secondly, the sharpness of the intra-f-shell emission lines of Eu(III) ions realizes pure red color, which is also advantageous for application in white LED devices. The ^5^D_0_→^7^F_2_ transition in Eu(III) is around 616 nm, and the luminosity factor is relatively large in the red region. This means that the effects of emission of Eu(III) complexes on the properties of the white spectra of LED devices are remarkable. Consequently, a noticeable improvement induced by the rare-earth ions is expected. On the other hand, the emission spectra of many red inorganic phosphors are broad, and the effects of longer wavelength components are small. Additionally, some spectra components are stretched to the near-infrared region and have no effect on the properties of LED devices.

Thirdly, when rare-earth complexes are dissolved in a polymer, colorless and transparent emission materials are obtained. They are invisible under daylight and emit beautiful pure color luminescence when they are irradiated by ultraviolet light, and have the potential to become ornaments.

Finally, the long luminescent lifetimes of Eu(III) ions, which are due to their forbidden intra-4f transition, unfortunately result in low absorption coefficients [11].

It is known that the emission of rare-earth ions can be enhanced through an indirect excitation mechanism. For Eu(III) complexes, β-diketonates are often used. Some of these diketonates absorb light and transfer energy to rare-earth ions. This transfer is efficiently followed by relatively high emission. Such diketonates are synthesized easily [12,13,14,15,16,17]. The emission intensity of Eu(III) complexes also depend strongly on the substituents of the β-diketonates, because the triplet-state energy levels are changed by their substituents.

However, it is difficult to excite rare-earth complexes efficiently enough for them to be used for emission devices by only adjusting the substituents of β-diketonates. The introduction of a photosensitizer, e.g., using 1,10-phenanthroline as ligand, is reported to be effective for exciting rare-earth ions [2,18].

Rare-earth complexes are known to be hard to dissolve in many of the polymers (or solvents) in large concentrations because they have high molecular weight and polarity. In particular, the solubility was drastically decreased with 1,10-phenanthroline, because this compound induces a strong molecular interaction by π stacking. 

Solvaton of the solute consists of two steps: the crystals of the solute are changed into amorphous solids, and solvated by solvent molecules. Generally, high molecular weight and polarity correlated to the first step and induced strong molecular interactions among solute molecules. Large amounts of energy are required to convert crystals into amorphous solids.

Introducing phosphine oxides in addition to β-diketonates to enhance the ligands field asymmetry, followed by the enlargening of the absorption coefficient, was reported [6,7,8,9,10]. The author focused on rare-earth complexes of this type because they have strong emission intensity and good solubilities. However, much larger emission intensity and greater solubility are prerequisites for the use of rare-earth complexes in many emission devices and diverse application fields.

## 2. Molecular Designs, Properties and Molecular Structures of Eu(III) Complexes with Two Different Phosphine Oxides

### 2.1. The concept of molecular designs of novel rare-earth complexes

**Figure 1:**
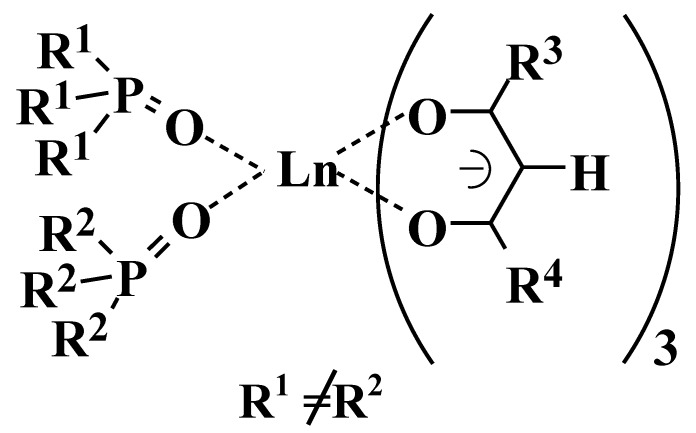
The first concept for the molecular design of a novel rare-earth complex with two different phosphine oxides.

The first concept for the molecular design of rare-earth complexes involves introducing two different phosphine oxides to one rare-earth ion, as shown in Figure 1 [19]. Non-symmetric molecular structures prevent the close packing of solute molecules and reduce the strength of intermolecular interactions. The decreasing symmetries of molecular structures and ligands fields are expected to be effective for improving the solubilities and emission intensities of the complexes.

However, ligand exchange of phosphine oxide ligands was detected by ^31^P-NMR analysis [20]. Ligand exchange was expected to have an undesirable effect on the durability and emission intensity of Eu(III) complexes. 

**Figure 2:**
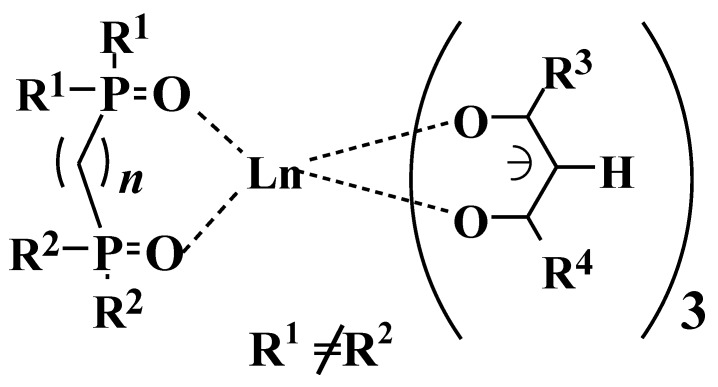
The second concept for the molecular design of novel rare-earth complex with non-symmetric bis-phosphine oxides.

The second molecular design involves two different phosphine oxides, which are tied together, and the introduction of a bis-phosphine oxide structure to a rare-earth ion in order to prevent ligand exchange, which is due to the chelate effect, as shown in Figure 2 [21]. 

### 2.2. Emission intensity of Eu(III) complexes with two different phosphine oxides

Emission intensity is a property that is proportional with both the absorption coefficient and the quantum yield of luminescent materials. Emission intensity is the index of performance for luminescent materials when they are irradiated by a fixed energy of light, and is a practical number for evaluating the potential of the usability of a complex for emission devices. When the excitation wavelength is settled on the maximum wavelength of LED chips, the relative emission intensity implies the performances of the luminescent materials when they are applied to the LED devices. 

Recently, LED devices with near-ultraviolet LED chips attracted much attention because they have a wide range of adjustment capacity for emission spectra due to the fact that near ultraviolet light is invisible. These chips can be applied to a variety of purposes. Because the maximum wavelength of the typical near-ultraviolet LED chips is around 395 nm, emission spectra were compared when they were excited by 395 nm light. Additionally, 395 nm is near the maximum absorption of the Eu(III) ion, indicating that there are two excitation processes for Eu(III) ions. Firstly, the ligands of Eu(III) complexes were excited by absorbing the light, and subsequently, energy transfer occurs from the ligand to the Eu(III) ion. The second process is that of direct absorption by the Eu(III) ion. Practical absorption consists of the above-stated two processes.

**Figure 3:**
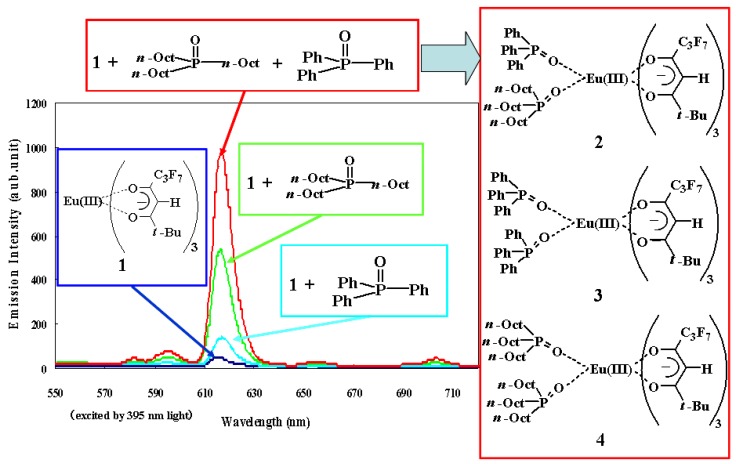
Emission spectra of the mixtures of Eu(III) complexes and phosphine oxides in a fluorinated solvent (2, 3-dihydrodecafluoropentane).

A dilute solution of Eu(III) complex **1** (2 × 10^−4^ M) and mixtures of **1** and triphenylphosphine oxide (TPPO) or trioctylphosphine oxide (TOPO), or both, in fluorinated solvent (2,3-dihydrodecafluoropentane) were prepared (Figure 3) [19], and their emission spectra, excited by 395 nm light, were compared.

In the case where an Eu(III) complex **1** was individually dissolved in fluorinated solvent, a very small emission intensity was observed. When the mixture of **1** and 2.0 molar equivalent of TPPO and/or TOPO was dissolved, the emission intensity was enlarged. Moreover, when the mixture of **1** and 1.0 molar equivalent of TPPO and TOPO was dissolved, the largest emission intensity was obtained. When the effects of introducing TPPO and TOPO are additive, the emission intensity of this mixture must be between those of the mixture of **1** and 2.0 molar equivalent of TPPO and the mixture of **1** and 2.0 molar equivalent of TOPO. Introducing two different phosphine oxides in one Eu(III) ion has special effects for improving emission intensity.

The quantum yield of the mixture of **1**, TPPO, and TOPO in the fluorinated solvent was 0.3 (excited at the maximum wavelength of excited spectra (335 nm), 6 × 10^−5^ M). It was also confirmed that quantum yields change little over the range from 1 × 10^−5^ to 2 × 10^−4^ M. Perfluoropropyl (C_3_F_7_), *t*-butyl (*t*-Bu), and *n*-Octyl groups are bulky, and it is expected that concentration quenching is prevented by them.

One reason that the largest emission intensity occurs with the mixture of **1**, TPPO, and TOPO is that the direct absorption of the Eu(III) ion is enhanced by asymmetric ligands field as a result of the introduction of two different phosphine oxides. 

**Figure 4:**
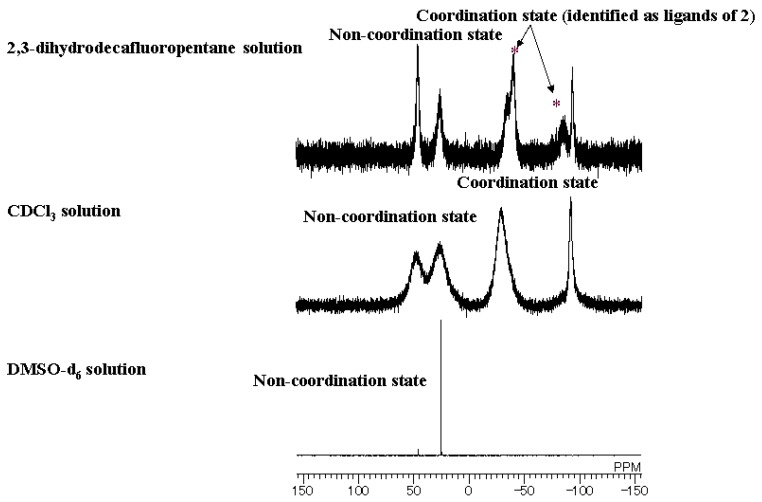
^31^P-NMR spectra (298 K) of a mixture of Eu(III) complex **1**, TOPO, and TPPO in fluorinated solvent, CDCl_3_, and DMSO-d_6_.

^31^P-NMR spectra of the mixture in 2,3-dihydrodecafluoropentane, CDCl_3_, and DMSO-d_6_ solutions at room temperature are shown in Figure 4 [20]. It was found that three Eu(III) complexes, **2**~**4**, are formed as a result of the mixing of the Eu(III) complex **1**, TPPO, and TOPO in the fluorinated solvent.

The signals corresponding to Eu(III) complex **2** are definitely identified in the fluorinated solvent. On the other hand, the spectrum of the mixture in CDCl_3_ is broader than that of the fluorinated solvent, and signals corresponding to Eu(III) complex **2** are not identified. The broader spectrum of the mixture in CDCl_3_ implies that the ligand exchanges are faster in CDCl_3_ than in the fluorinated solution. The signals of phosphine oxides acting as ligands were not detected at all in DMSO-d_6_ solution, indicating that no phosphine oxide was coordinated with an Eu(III) ion. In general, the thermodynamic stabilities of ternary complexes are solvent-dependent. The more polar the solvent, the less stable the complex. DMSO is very polar. Moreover, DMSO strongly coordinates to rare-earth cations, which prevents phosphine oxides from coordinating.

When phosphine oxides exist as ligands, the signals have been reported to shift to upper magnetic fields [22]. DMSO-d_6_ is a strong Lewis base, and thus, DMSO-d_6_ solution contains an excess of DMSO-d_6_ molecules, which act as ligands, making it difficult for the phosphine oxide molecules to coordinate with Eu(III) ions. In DMSO solution, TPPO cannot coordinate with an Eu(III) ion. However, TOPO is stronger Lewis base than TPPO is, and part of the TOPO molecules are considered to coordinate with an Eu(III) ion. Because the bond length between an oxygen atom of a phosphine oxide and an Eu(III) ion is longer than that of other solvents (due to the effect of very polar and coordinative DMSO molecules), the signal of coordinated TOPO can be too broad to be clearly detected.

**Figure 5:**
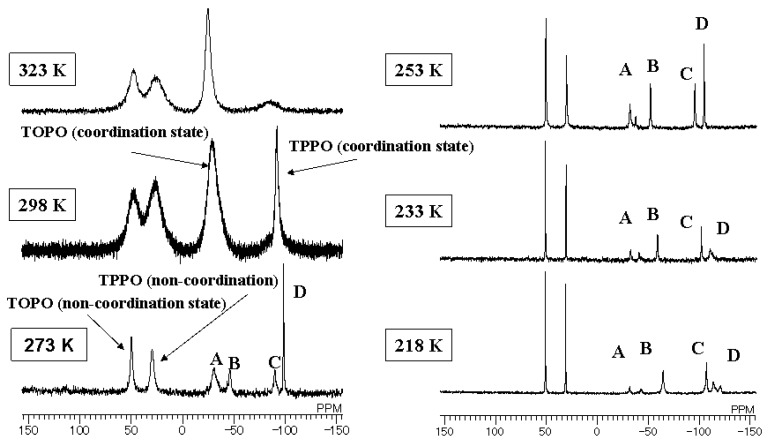
Temperature dependence of ^31^P NMR spectra of a mixture of Eu(III) complex **1** and two phosphine oxides in CDCl_3_ (A relates to TOPO in **4**, D relates to TPPO in **3**, B relates to TOPO in **2**, and C relates to TPPO in **2**).

As shown in Figure 5, the temperature dependence of ^31^P NMR spectra of the mixtures of Eu(III) complexes in CDCl_3_ was investigated. The sharpness of the signals can be observed to increase as measurement temperature decreases. This is thought to be due to the slowing down of ligand exchanges at low temperatures. Below 273 K, signals corresponding to Eu(III) complex **2** (B and C in Figure 5) were observed. The intensity of these signals compared to those of complexes **3** and **4** were evidently lower in the case of fluorinated solvent (even considering that the intensity of ^31^P NMR spectra are not quantitative). Moreover, in CDCl_3_ solution, the strengths of signals B and C increased with the decrease in temperature.

The coordination ratio of TPPO and TOPO in the solution of Eu(III) complex **1** is considered to be influenced by the phosphine oxides’ solubilities. When phosphine oxides are highly soluble in a solvent, the equilibrium shifts from the coordination state toward the non-coordination state. When phosphine oxides are less soluble in a solvent, the equilibrium shifts to former. Generally, polar compounds, such as phosphine oxides, are highly soluble in polar solvents and less soluble in hydrophobic fluorinated solvents. This means that larger ratios of TPPO and TOPO are coordinated with an Eu(III) ion in a hydrophobic fluorinated solvent compared with that of a polar hydrophilic solvent. 

On the other hand, ligand exchanges of phosphine oxides in Eu(III) complexes were observed by NMR analysis [20] in the mixture of Eu(III) complexes **1**, TPPO, and TOPO. These results indicate that the product ratios of Eu(III) complexes **2**, **3**, and **4** in the solutions are considered to be influenced by both the solvents and solubilities of produced complexes. In the fluorinated solvent, a larger majority of TPPO and TOPO tend to coordinate with an Eu(III) ion, and the highly soluble complexes are considered to be produced preferentially, so that the sum of the concentrations of Eu(III) complexes with two phosphine oxides, **2**, **3**, and **4**, are maximized. The Eu(III) complex with two different phosphine oxides (complex **2**) is highly soluble compared with complexes with two of the same phosphine oxides (complexes **3** and **4**) and is produced preferentially in the fluorinated solvent. In the CDCl_3_ solutions, TPPO and TOPO are more soluble compared with those in fluorinated solvent and their ratios of coordination state are small. Produced complexes **2**, **3**, and **4** are considered to be all highly soluble in CDCl_3_ compared with their solubility in the fluorinated solvent.

Above all, the ratio of Eu(III) complex **2** to the sum of the produced Eu(III) complexes **2**, **3**, and **4** in the mixture of Eu(III) complex **1**, TPPO, and TOPO in fluorinated solvents is larger than that of CDCl_3_. This prediction is confirmed by the observed greater intensity of the signals of Eu(III) complex **2** in the fluorinated solvent than in CDCl_3_. The largest emission intensity of the mixture of Eu(III) complex **1**, TPPO, and TOPO in fluorinated solvent is due to the largest production ratio of complex **2**.

TPPO and TOPO are highly soluble, even at low temperatures, in CDCl_3_. On the other hand, the solubilities of complexes **2**~**4** were decreased. As a result, equilibrium of TOPO and TPPO between coordination state and non-coordination state in complexes **3** and **4** shifts toward the latter state at low temperatures. This induces the smaller spectra intensities. The intensities of signals B and C decrease little even at low temperatures because temperature decrease has little effects on the solubility of Eu(III) complex 2 with two different phosphine oxides.

**Figure 6:**
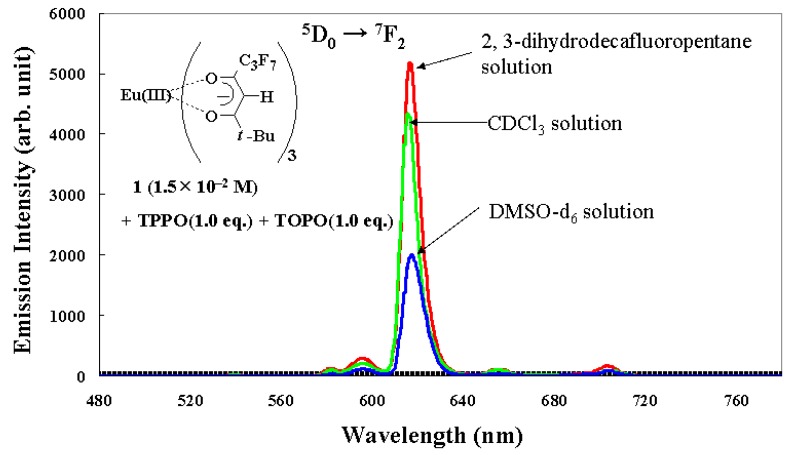
The emission intensities of a mixture of Eu(III) complex **1** (1.5 × 10^−2^ M), TOPO (1.0 equation), and TPPO (1.0 equation) in fluorinated solvent (2,3-dihydrodecafluoropentane), CDCl_3_, and DMSO-d_6_ excited at 395 nm at room temperature.

Figure 6 shows the relative emission intensities of the mixture of Eu(III) complex **1**, TOPO, and TPPO, in fluorinated solvent, CDCl_3_, and DMSO-d_6_, excited at 395 nm, which is the maximum wavelength supported by our ultraviolet LED chip [20]. The highest emission intensity was obtained in the fluorinated solvent. This is ascribed to the prediction that highly soluble Eu(III) complex **2** with two different phosphine oxides are predominantly formed and the ratio of complex **2** to sum of the produced Eu(III) complexes **2**, **3**, and **4** in the mixture of Eu(III) complex **1**, TPPO, and TOPO becomes large. Slow ligand exchanges of phosphine oxides detected by NMR analysis contribute to improving emission intensity.

In CDCl_3_ solution, a relatively large emission intensity was obtained. On the other hand, only a low emission intensity was obtained in DMSO-d_6_ solution. The emission intensity of Eu(III) complex **1** in DMSO-d_6_ is not changed when one molar equivalent of TPPO and TOPO are added to the solution. Additionally, the emission lifetime of complex **1** is 7.9 × 10^−4^ s and is almost the same as that of the mixture of complex **1**, TPPO, and TOPO in DMSO-d_6_ solution (7.6 × 10^−4^ s). These results indicate that a very small amount of phosphine oxides is coordinated with Eu(III) ions or that ligand exchanges of phosphine oxide are very fast, or both. The combination of Eu(III) complexes with two different phosphine oxides and the fluorinated solvent provides the highest emission intensity.

### 2.3. Solid-state ^31^P NMR analysis of Eu(III) complexes with phosphine oxides in fluorinated polymer

**Figure 7:**
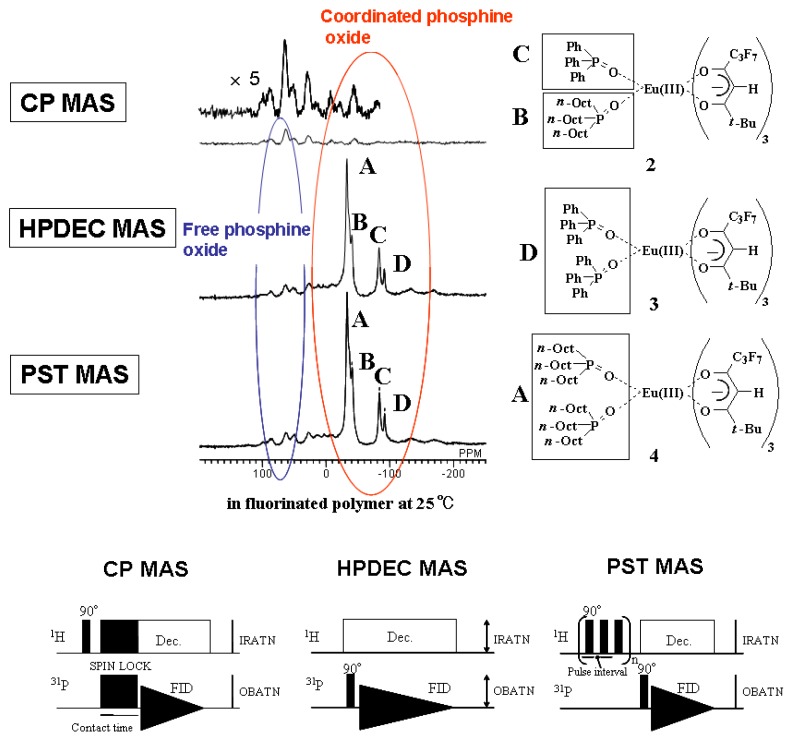
Comparison of three types of solid-state ^31^P NMR spectra of the mixture of Eu(III) complex **1**, TOPO, and TPPO.

Three types of solid-state ^31^P NMR analyses were carried out on the mixture of Eu(III) complex **1**, TOPO, and TPPO in fluorinated polymer consisting of a fluorinated polymer backbone and acrylic polymer branches: cross-polarization and magic-angle spinning (CP MAS), pulse-saturation transfer and magic-angle spinning (PST MAS), and gated high-power decoupling and magic-angle spinning (HPDEC) analysis [23]. In a manner similar to solution NMR analysis in the case of PST MAS, the signals of the observed nuclei are enhanced by the Nuclear Overhauser Effect (NOE). This measurement method is more effective for nuclei that are not restricted in their movement, and the signals of areas where nuclei are located with high degrees of molecular motion, such as the amorphous parts of polymers, are enhanced. On the other hand, in CP MAS analysis, the nuclear spin of an atom whose molecular motion is restricted will be enhanced, because a shift in magnetization occurs through dipole interactions. HPDEC MAS analysis is the quantitative ^31^P NMR analysis. Figure 7 shows the solid-state ^31^P NMR spectra of the mixture obtained by the three aforementioned methods [23].

In the case of PST MAS and HPDEC MAS analysis, four signals, which shift to upper magnetic fields from the original chemical shifts of TOPO and TPPO, are distinctly observed. They correspond to the signals of coordinated phosphine oxides. Three Eu(III) complexes **2**~**4** were identified and their signals are clearly distinguishable at room temperature. On the other hand, in the case of the CP MAS spectrum, no clear signal was obtained. 

The results that signals of coordinated phosphine oxides were observed in PST MAS and HPDEC MAS analysis and not observed in CP MAS analysis indicate that phosphine oxides coordinated with an Eu(III) ion have a high degree of molecular motion. This means that Eu(III) complexes with two phosphine oxides are not uniformly dissolved in fluorinated polymer and are assumed to exist mainly in the flexible amorphous parts of the fluorinated polymer, not in the rigid crystallized parts. Amorphous polymers are considered to be suitable as a matrix for Eu(III) complexes because a higher concentration of complexes can be dissolved and a larger emission intensity is expected.

### 2.4. The solubilities in fluorinated medium, melting points, and thermodynamic properties of Eu(III) complexes with phosphine oxides

**Figure 8:**
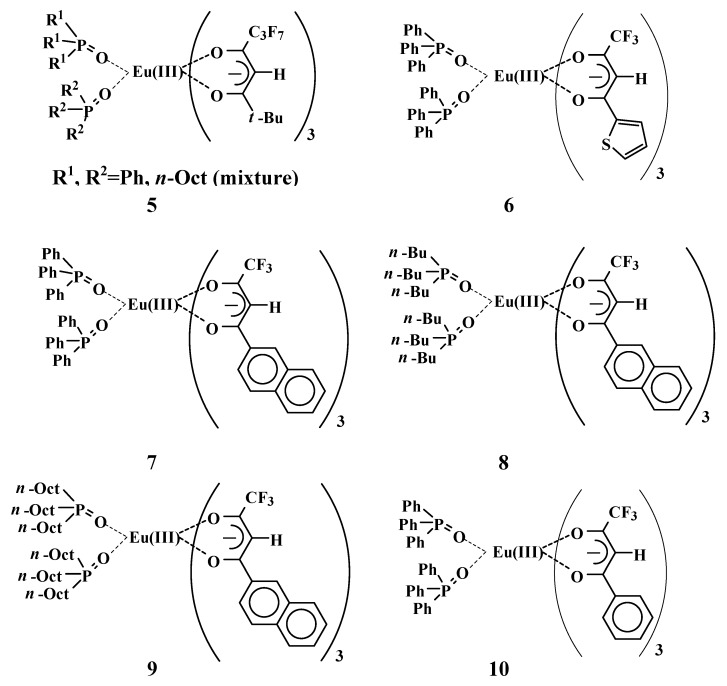
Eu(III) complexes with β-diketonates and phosphine oxides used in this study.

Correlations among the molecular structures of **5**~**10** (Figure 8), solubilities, melting points, and thermodynamic properties were investigated (Table 1) [24].

**Table 1:** Solubilities, melting points, and thermodynamic properties of Eu(III) complexes with β-diketonates and phosphine oxides.

		**Thermodynamic Property**		**Solubility (μ mol/g)**	
	*T*_m_^1^	Δ*H*_f_^2^	Δ*S*_f_^3^	hexane	2,3-dihydrodecafluoropentane
**5**	377	2.5	0.0066	211	8.6
**6**	441	83	0.19	0.0027	<0.001
**7**	427	76.9	0.18	0.013	0.0039
**8**	352	37	0.11	92.1	0.052
**9**	353	101	0.29	19.7	<0.001
**10**	412	39.9	0.097	0.079	<0.001

^1^ melting point (K); ^2^ enthalpy of fusion at melting point (kJ/kg); ^3^ entropy changes (kJ/kg K)

Eu(III) complex **5**, with two different phosphine oxides, has a low *T*_m_ and a small Δ*H*_f_. It is highly soluble in both hexane and in the fluorinated solvent compared with other complexes **6**~**10**. Complex **5** has a bulky perfluoroalkyl group, a *t*-Bu group in β-diketonates, and a non-symmetric molecular structure induced by two different phosphine oxides. 

Flexible polar parts of the solute are effective for improving solubility because they easily form suitable conformations to enlarge the interactions with polar parts of solvent molecules [25,26,27]. 

Eu(III) complex **9** has two flexible and hydrophobic TOPO (6 *n*-octyl units), but its solubility is very small (<0.001). The introduction of two different phosphine oxides is considered to be the main factor for improving the solubility from the results that are listed in Table 1. 

### 2.5. Eu(III) complexes with β-diketonates and a non-symmetric bis-phosphine oxide

**Figure 9:**
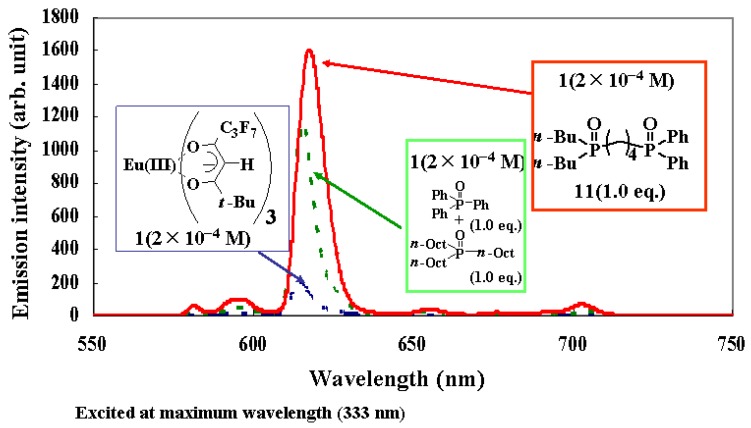
Emission spectra of the mixtures of Eu(III) complex **1** and phosphine oxides in the fluorinated solvent (2, 3-dihydrodecafluoropentane).

Non-symmetric bis-phosphine oxide **11** was synthesized and mixed with 1.0 molar equivalent of Eu(III) complex **1** in fluorinated solvent, as shown in Figure 9 [21]. The emission spectrum of the solution is shown in Figure 9, together with that of a mixture of **1** and 1.0 molar equivalent of TPPO and TOPO. The emission intensity of the mixture of **1** and **11** is larger than that of the mixture of **1**, TPPO, and TOPO.

**Figure 10:**
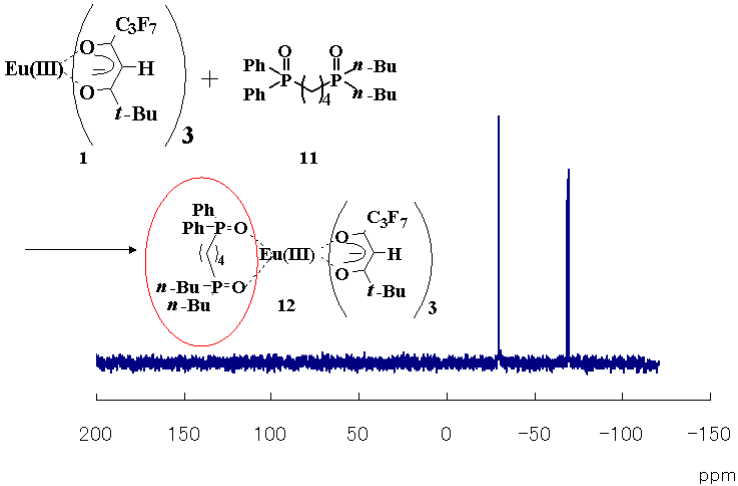
^31^P NMR spectrum of the mixture of Eu(III) complex **1** and non-symmetric bis-phosphine oxide **11**.

The signals of the ^31^P NMR spectrum for the mixture of Eu(III) complex **1** and non-symmetric bis-phosphine oxide **11** are very sharp and are shifted toward upper magnetic fields from the original ones of **11**, suggesting that all the non-symmetric bis-phosphine oxide **11** coordinated with Eu(III) ion without ligand exchange, and that Eu(III) complex **12** was formed in the solution (Figure 10) [21]. The larger emission intensity of the mixture of **1** and **11** shown in Figure 9 is due to the formation of Eu(III) complex **12**, in which two different phosphine oxide structures are coordinated perfectly to one Eu(III) ion.

**Figure 11:**
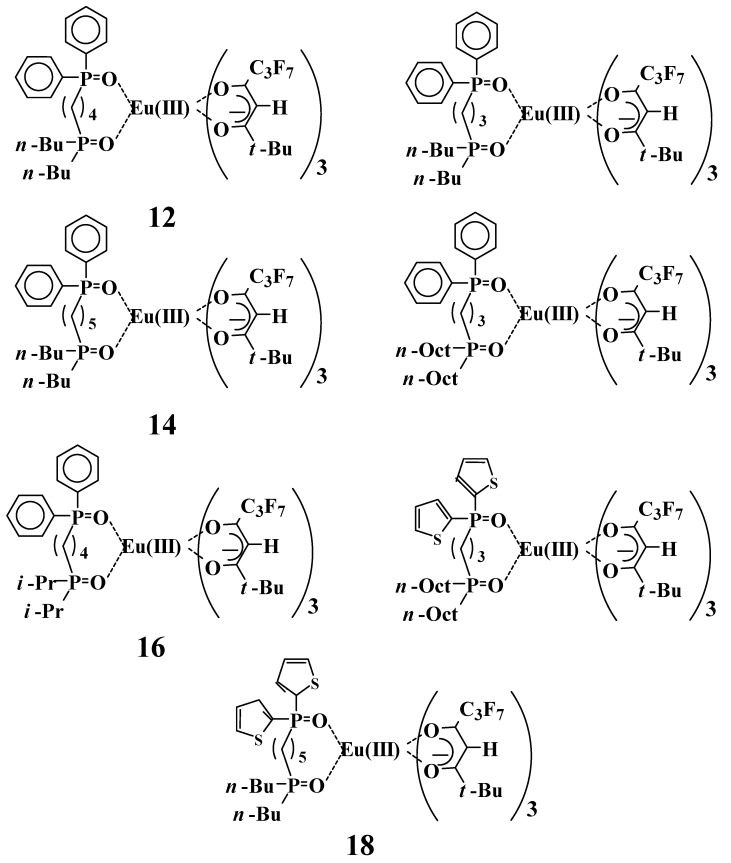
Molecular structures of Eu(III) complexes with non-symmetric bis-phosphine oxides.

Several novel Eu(III) complexes with non-symmetric bis-phosphine oxides were synthesized (Figure 11) and their melting points, thermodynamic properties, and solubilities in 2,3-dihydrodecafluoropentane were investigated (Table 2) [21]. The sum of the enthalpy of fusion and phase transition (Δ*H*_(SUM)_) is the heat capacity that is required to change the definite amount of crystals of an Eu(III) complex located at room temperature into amorphous solid; this sum, from the results of dichroic dyes, is known to be correlated with the solubility [25,26].

Thermodynamic properties were correlated to the molecular structures and solubility of the solute. A small sum of the enthalpy of fusion and phase transition (Δ*H*_(sum)_) and a low melting point (*T*_m_) are preferable for high solubility. Eu(III) complexes **12**, **13**, and **14** have two of the same substituents (Ph and *n*-Bu groups), and only “*n*” (the length of alkyl chain that binds two phosphine oxide parts) is different. Among these, the *T*_m_ of the complexes **13** (*n* = 3) and **14** (*n* = 5) were remarkably lower than that of complex **13** (*n* = 4), and the Δ*H*_(sum)_ of **13** and **14** were smaller than that of **12**. Additionally, the solubilities of **13** and **14** are much larger than that of **12** (correlated to the results of thermodynamic properties). Complexes **12** and **16** (*n* = 4) have a high *T*_m_ and a large Δ*H*_(sum)_, and the solubilities are relatively small compared with those of other complexes. These results suggest that the steric effects of coordination structures are dependent on the “*n*” and have evident effects on thermodynamic properties and solubility. Complexes **17** and **18**, by virtue of having thienyl groups, are relatively highly soluble when compared with the solubilities of other complexes.

**Table 2:** The solubilities, melting points, and thermodynamic properties of Eu(III) complexes with β-diketonates and phosphine oxides.

**Complex**		**Thermodynamic properties**				**Solubility ^5^**
	*T*_m_^1^	Δ*H*_f_^2^	Δ*H*_tr_1 ^3^	Δ*H*_tr_2 ^3^	Δ*H*_(SUM)_^4^	
	(K)	kJ/kg(K)				Eu:μ mol/g
**12**	507	44.0	3.09 (465)	5.59 (490)	52.68	0.02
**13**	443	37.5	-	-	37.5	0.72
**14**	462	27.0	90.49 (366)	11.1 (392)	38.59	0.55
**15**	389	1.27	21.3 (341)	1.93 (381)	24.5	1.2
**16**	453	34.5	2.97 (406)	-	37.47	0.47
**17**	372	7.54	11.5 (350)	-	19.04	2.2
**18**	419	14.0	4.83 (353)	2.20 (364)	21.03	2.5

^1^ melting point; ^2^ enthalpy of fusion at melting point; ^3^ enthalpy change at phase transition; ^4^ sum of the enthalpy of fusion and phase transition; ^5^ measured in 2,3-dihydrodecafluoropentane at room temperature.

The evident negative correlation between the solubilities of Eu(III) complexes and Δ*H*_(SUM)_ are observed. Correlations among molecular structures, melting points, thermodynamic properties, and solubilities are explained below. Non-symmetric molecular structures induced by non-symmetric bis-phosphine oxide ligands prevent close packing, and reductions of melting points and the energies of molecular interaction (Δ*H*_(SUM)_) are followed by increases in solubilities.

### 2.6. Emission properties of Eu(III) complexes with a non-symmetric bis-phosphine oxide

The emission intensity of the mixture of Eu(III) complex **1**, 1.0 equivalent of TPPO, and TOPO in 2,3-dihydrodecafluoropentane at the concentration of 2 × 10^−4^ M is 1202 (arbitrary unit) (Figure 9). On the other hand, the emission intensities of many Eu(III) complexes with non-symmetric bis-phosphine oxides in the same condition (Table 3) are larger than the value of 1202. When comparing the emission intensities of complex **15** (1607, arb. unit) with that of the mixture of **1**, TPPO, and TOPO, it was found that the intensity of complex **15** is 1.3-fold of that of the latter mixture. 

The difference of molecular structures between complexes **15** and **5**, which is the main component in the mixture, is whether phenyl phosphine oxide and octyl phosphine oxide ligands are together or not. These results indicate that the introduction of non-symmetric bis-phosphine in the Eu(III) complex is effective for improving emission intensity.

The emission intensity of complex **16** is relatively smaller than those of other complexes. Branched chain *i*-propyl groups prevent the complex from forming a steric coordination structure with a large emission intensity, due to the bulky structure near the P=O groups or the stronger electrodonating properties, or both. 

On the other hand, complexes **17** and **18**, which have thienyl groups instead of phenyl groups, have relatively smaller emission intensities. To clarify the reason for the smaller emission intensities of complexes **17** and **18**, quantum yields of complexes **12**~**18** in CCl_4_ solution were investigated. Emission intensities are proportional to the products of absorption coefficients and quantum yields; intensities are reduced by vibration-inactivation. C-H bonds are known to act as effective vibration-inactivation bonds, and it is expected that 2,3-dihydrodecafluoropentane reduces the emission intensity. CCl_4_ was selected as a solvent in the measurements of quantum yields because there is no possibility of radiationless transition. The effect of vibration-inactivation can be ignored in the solvent.

Quantum yields of complexes **12**~**16** were satisfactory and with no great differences between them (0.45~0.52). On the other hand, the quantum yields of complexes **17** and **18** are relatively smaller (0.38 and 0.34, respectively) than those of complexes **12**~**16**. Complexes **17** and **18** have thienyl groups in non-symmetric phosphine oxides instead of phenyl groups, and it is expected that this causes low quantum yields.

**Table 3:** The emission intensity and quantum yields of Eu(III) complexes with β-diketonates and non-symmetric bis-phosphine oxides.

Complex	Emission intensity (arb. unit) ^1^	Quantum yields ^2^
**12**	1588	0.52
**13**	1676	0.47
**14**	1836	0.46
**15**	1607	0.49
**16**	1168	0.45
**17**	1467	0.38
**18**	1507	0.34

^1^ measured in 2,3-dihydrodecafluoropentane at a concentration of 2 × 10^-4^ M and excited at maximum wavelength; ^2^ measured in CCl_4_ at a concentration of 4 × 10^-4^ M and excited at maximum wavelength

**Figure 12:**
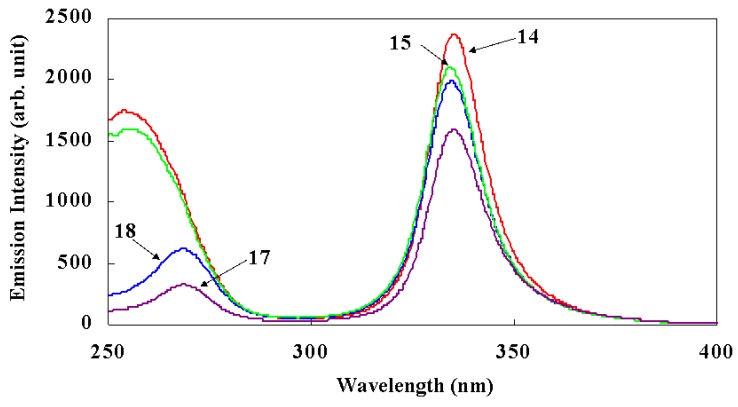
Comparison of the excitation spectra of Eu(III) complexes **17** and **18**, which have thienyl groups in non-symmetric phosphine oxides, with the spectra of complexes **14** and **15**, which have phenyl groups (measured in CCl_4_, 2 × 10^−4^ M, room temperature, excited at 336 nm light).

To clarify the introduction of thienyl groups in non-symmetric bis-phosphine oxides, excitation spectra of complexes **17** and **18** were compared with complexes **15** and **14**, respectively (Figure 12). The molecular structures of complexes **17** and **15** are the same, with the exception of thienyl groups (**17**) and phenyl groups (**15**). Similarly, the molecular structures of complexes **18** and **14** are the same, with the exception of thienyl groups (**18**) and phenyl groups (**14**).

Each emission spectrum has two peaks. With respect to the peaks of longer wavelengths, the emission intensities of Eu(III) complexes **15** and **14** with phenyl groups in non-symmetric phosphine oxides are found to be evidently larger than their respective counterparts, as measured from complexes **17** and **18**, which possess thienyl groups. These results are consistent with the values of quantum yields measured at the maximum wavelength of longer wavelength peaks. On the other hand, the maximum wavelengths in the shorter wavelength region of complexes **17** and **18** are longer than those of complexes **14** and **15**, and emission intensities are drastically reduced. This wavelength region corresponds to absorption by bis-phosphine oxides. The efficiency of energy transfer from the excited states of bis-phosphine oxides to the *5d* orbital of Eu(III) is reduced by the thienyl group.

As a consequence, phenyl groups are more preferable than thienyl groups for improving large emission properties.

### 2.7. Tb(III) complexes with two different phosphine oxides and their properties *[28]*

**Figure 13:**
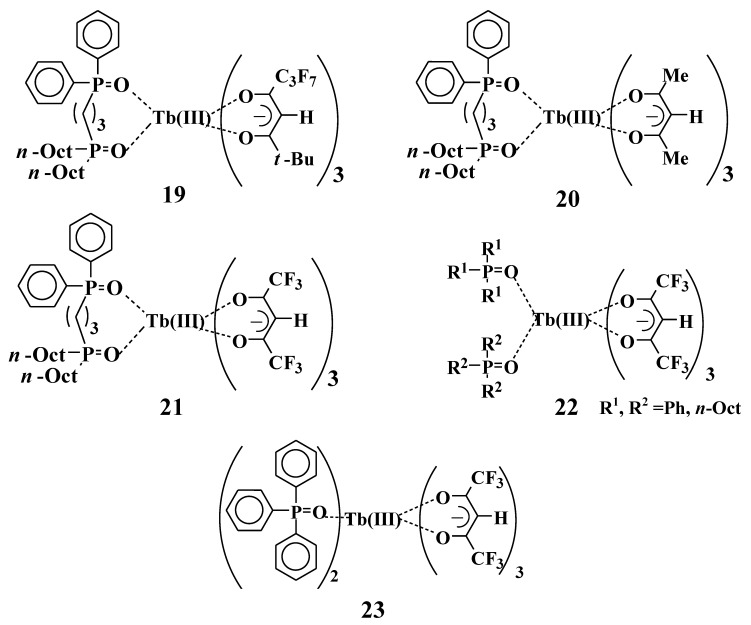
The molecular structures of Tb(III) complexes with the phosphine oxides used in this study.

Recently, Tb(III) complexes have emerged as promising candidates for a thermosensor because their emission intensities are drastically changed by temperature [29,30,31]. However, the solubility and emission intensity of Tb(III) complexes are much smaller than those of Eu(III) complexes, and further improvement is required for many applications. The molecular structure of bis-phosphine oxide is designed to be non-symmetric in a way that is analogous with the Eu(III) complexes. The structures have two different phosphine oxides so as to attain large solubilities and emission intensities. Four Tb(III) complexes with one non-symmetric bis-phosphine oxide and three β-diketonates **19**~**22** (Figure 13) were synthesized [28].

**Figure 14:**
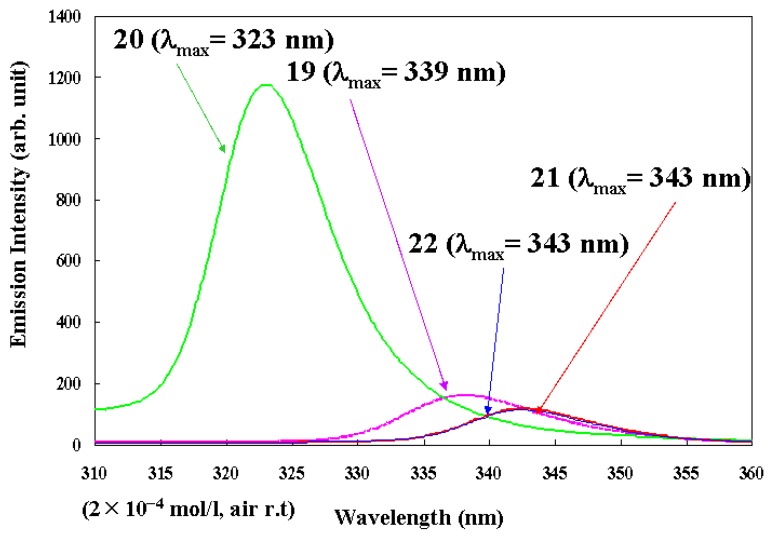
Excitation spectra of Tb(III) complexes **19**~**22** (2 × 10^−4^ M) in ethyl acetate.

The excitation spectra of Tb(III) complexes **19**~**22** (2 × 10^−4^ M) are shown in Figure 14. The measured maximum wavelengths (λ_exp_) are clearly correlated to the substituents of the β-diketonate ligands. In the case that two substituents are electron-donating groups (e.g., methyl groups in complex **20**), λ_exp_ is the shortest, and emission intensity at the λ_exp_ (*I*_exp_) is the largest. When the two substituents are electron-attractive groups (e.g., trifluoromethyl groups (CF_3_) in complex **21**), λ_exp_ is the longest and *I*_exp_ is the smallest. In the case that β-diketonates have one electron-donating group and an electron-attractive group (e.g., C_3_F_7_ and *t*-Bu groups in complex **19**), both λ_exp_ and *I*_exp_ are between those of complexes **20** and **21**, respectively. On the other hand, when comparing the excitation spectra of complexes **21** and **22**, the differences with respect to whether two phosphine oxide parts are tied together or not have little effect on λ_exp_ and *I*_exp_.

The calculated λ_cal_, λ_exp_, and Δ*E*_cal_ of each ligand are summarized in Table 4. It can be found that the λ_cal_ of three types of β-diketonates are in the same order as the λ_exp_ in 1 × 10^−3^ M solution of ethyl acetate, and that differences between λ_cal_ and λ_exp_ are small.

The pronounced correlation between λ_exp_ and *I*_exp_ shown in Figure 14 is explained using both the calculated and the experimental results mentioned below. Shorter wavelength shifts of λ_exp_ in Figure 14, when electron-donating groups are substituted in β-diketonates, lead to larger Δ*E*_cal_ values. When Δ*E*_cal_ values are larger, back energy transfers from excited Tb(III) ions to the ligand [2,12] are restrained; this effect enlarges the quantum yields, and consequently, there are larger *I*_exp_ values (Table 3).

The emission lifetime and emission quantum yield of Tb(III) complex **20**, which has the largest *I*_exp_ in Figure 14, are *τ*_obs_ = 0.89 ms (excited at 335 nm) and Φ = 0.15 (excited at 335 nm) in dilute solution (1 × 10^−3^ M) of CCl_4_ at room temperature, respectively. The calculated radiation rate (emission quantum yield / emission lifetime) of Tb(III) complex **20** is about 170. On the other hand, in the case of complex **19**, which has a lower *I*_exp_, the emission lifetime and emission quantum yield are *τ*_obs_ = 0.05 ms (excited at 365 nm) and Φ = 0.04 (excited at 355 nm). This marked difference between the properties of complexes **19** and **20** is considered to be correlated to the back energy transfer. 

**Table 4:** The calculated maximum wavelength (λ_cal_), measured maximum wavelength in 1 × 10^−3^ M solution of ethyl acetate (λ_exp_), energy difference between the ^5^D_4_ level of Tb(III) and the lowest triplet-state energy level of the ligand (Δ*E*_cal_ (eV)), and intensity of excitation spectra at λ_exp_ in 1 × 10^−3^ M solution of ethyl acetate (*I*_exp_). 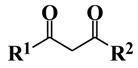

R^1^	R^2^	As ligand	λ_cal_ (nm)	λ_exp_ (nm)	Δ*E*_cal_ (eV)	*I*_exp_
Me	Me	Ligand of complex **20**	319	323	0.75	1200
*t*-Bu	C_3_F_7_	Ligand of complex **19**	334	339	0.47	180
CF_3_	CF_3_	Ligand of complex **21** and **22**	337	343	0.31	120

### 2.8. Emission properties of Tb(III) complexes

**Figure 15:**
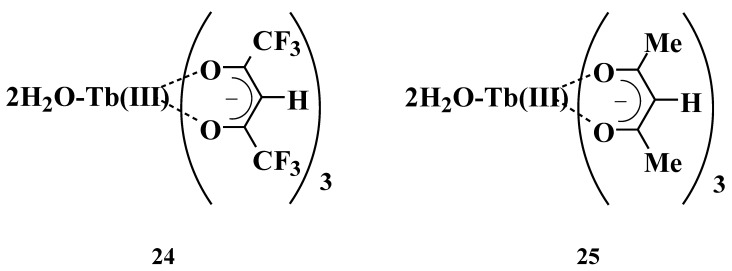
Molecular structures of Tb(III) complexes without phosphine oxide.

**Figure 16:**
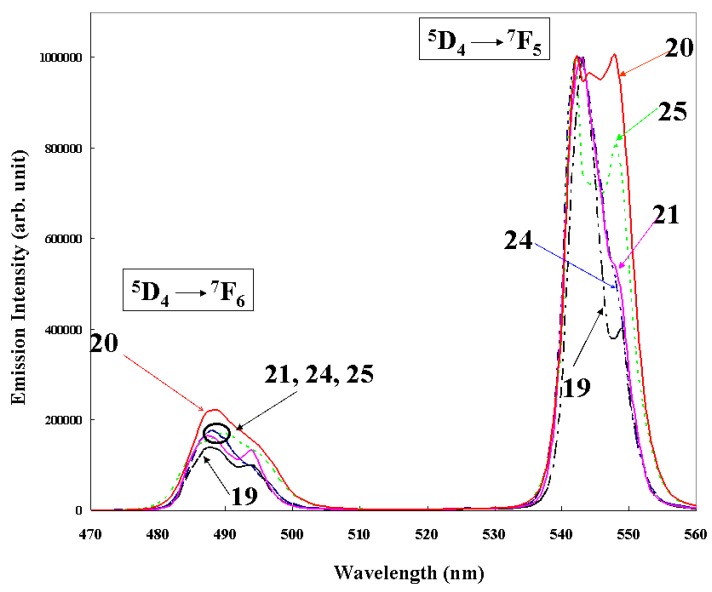
The emission spectra of Tb(III) complexes **19**, **20**, **21**, **24**, and **25** in ethyl acetate (normalized with respect to the transition “from ^5^D_4_ to ^7^F_5_”, which corresponds to the magnetic-dipole transition).

The referential peaks chosen were “^5^D_4_ to ^7^F_5_” (543 nm) and “^5^D_4_ to ^7^F_6_” transitions (488 nm), which correspond with the magnetic-dipole transition “from ^5^D_4_ to ^7^F_5_” of Δ*J* = 1, and the electric-dipole transition (from ^5^D_4_ to ^7^F_6_) of Δ*J* = 2, respectively. Tb(III) complexes, **24** and **25**, without phosphine oxide ligand (Figure 15), were prepared and their emission spectra were compared with those of Tb(III) complexes with two different phosphine oxides to explain the effects of their introduction. The emission intensities of Tb(III) complexes **19**, **20**, **21**, **24**, and **25** are shown in Figure 16. The spectra were normalized with respect to the “from ^5^D_4_ to ^7^F_5_” transition, which corresponds to the magnetic-dipole transition, being independent of the ligands fields. The branching ratio is defined as that of the intensity of electric-dipole transition “from ^5^D_4_ to ^7^F_6_” to the magnetic-dipole transition “from ^5^D_4_ to ^7^F_5_” [6]. Asymmetry of the ligand field is reflected as an increase in the branching ratio and is represented in Figure 16 as the intensity of electric-dipole transition.

Tb(III) complex **20** has the largest emission intensity of electric-dipole transition, suggesting that complex **20** has the ligand field symmetry despite the fact that the two substituents of β-diketonates are the same (methyl(Me) groups). On the other hand, the intensity of electric-dipole transition of complex **19** with two different substituents (*t*-Bu and C_3_F_7_ groups) on β-diketonate is the smallest. The intensities of electric-dipole transitions in complexes **21**, **24**, and **25** are between those of complexes **19** and **20**, and are almost the same. It is noted that introduction of two different substituents of β-diketonates has little effect on increasing the ligand field asymmetry. The steric effects of the substituents of β-diketonates are correlated with the ligands fields instead. Namely, complex **20** has two small methyl groups; the branching ratio of complex **20** is the largest. On the other hand, complex **19** has two different bulky substituents on β-diketonates; the branching ratio is the smallest. The substituent of β-diketonates in complexes **21** and **24** is the CF_3_ group, whose steric effects are between those of complexes **19** and **20**, and their branching ratios are also between those of complexes **19** and **20**. In the case of complex **20**, the bond lengths of oxygen atoms in phosphine oxides or β-diketonates, or both, and a Tb(III) ion are considered to be smaller than those of complex **19** because of the weaker steric effects that induce the ligand field asymmetry.

If complexes **20** and **25** are compared, it is evident that the introduction of phosphine oxide ligands enlarges the branching ratio, indicating that the ligand field becomes less symmetric. On the other hand, if complexes **21** and **24** are compared, introduction of phosphine oxide ligands has little effect on branching ratio, which suggests that ligands field is not changed. Bulky substituents of β-diketonates are considered to interfere with the changing of the steric coordination structure.

### 2.9. Solubilities of Tb(III) complexes

The solubilities of Tb(III) complexes in 2,3-dihydrodecafluoropentane (fluorinated solvent) are shown in **Table 5**. 2,3-dihydrodecafluoropentane was selected as a solvent for the measurements of solubilities because it is useful for predicting which rare-earth complexes are dissolvable in a fluorinated polymer at supersaturated high concentrations [24].

**Table 5:** The solubility of Tb(III) complexes used in this study.

Complex	Solubility (μ mol/g)
**19**	1.1
**20**	0.018
**21**	0.63
**22**	3.6
**23**	0.6

In general, although high-polar rare-earth complexes can be dissolved in a polar solvent, such as alcohol, they do not dissolve easily into many organic solvents, and this tendency is particularly marked in the case of strongly hydrophobic ones. Among hydrophobic solvents, compared with hexane, it was found to be very hard for 2,3-dihydrodecafluoropentane to dissolve Eu(III) complexes [24]. However, among the solvents, emission intensity of Eu(III) complexes was the highest when dissolved in a 2,3-dihydrodecafluoropentane, indicating that a fluorinated polymer is suitable for obtaining high emission [20]. Rare-earth complexes are dissolved into many kinds of solvents and polymers with high concentrations for use in various applications.

Solubility is clearly correlated with the substituents of β-diketonates when compared with Tb(III) complexes **19**~**21**, which have the same structures, except for the said substituents. Complex **19** has bulky substituents, which are *t*-Bu and C_3_F_7_ groups; it is highly soluble when compared with other complexes. The solubility of complex **21**, which has CF_3_ groups, is higher than that of complex **20**. The steric effects of substituents for β-diketonates are effective for increasing solubility. Complex **22** was synthesized for comparison with **21** to clarify the chelating effect of phosphine oxide ligands. The difference in the molecular structures of complexes **21** and **22** is that an octylphosphine oxide part and a phenylphosphine oxide part are tied together in the case of complex **21**, but they coordinate individually in the case of complex **22**. The solubility of complex **22** is six-fold greater than that of complex **21**, indicating that degree of freedom of steric coordination structure is important for improving solubility.

Tb(III) complex **23**, with two TPPO, was prepared [29] for comparison with complex **22** to clarify the effect of having two different phosphine oxides. Solubility of complex **22** is six-fold greater than that of complex **23**. The introduction of two different phosphine oxides to one Tb(III) ion is found to be remarkably effective for increasing solubility in the same way as seen in Eu(III) complexes [24].

The solubilities of rare-earth complexes in the liquid media shown in Table 5 are very small. In many application fields, rare-earth complexes are dissolved in polymers. In particular, when they are used as fluorescent compounds in LED devices, very large concentrations of complexes are required. 

It is expected that some properties of rare-earth complexes in solid media are different from what is observed when the complexes are in liquid media. In solid media, the amorphousness of rare-earth complexes plays a very important role. Generally, highly soluble compounds are highly amorphous. Rare-earth complexes dissolved in a fluorinated polymer of LED devices [19] exist in a state of supersaturation. The synergism of solubility effects and the complex’s amorphousness allows the complex to exist in a state such that large concentrations of rare-earth complexes are dissolved in a polymer. Emission lifetimes of rare-earth complexes are also influenced by the states of the solvents. The emission lifetimes of Tb(III) complex **22** are 0.75 ms (in ethyl acetate), 0.89 ms (in carbon tetrachloride), and 0.69 ms (in acrylic resin). The details of correlations among emission lifetimes, molecular structures, and solvents (liquid or solid state) are now under progress.

### 2.10. Density functional theory calculations of Eu(III) complexes with three β-diketonates and two phosphine oxides

**Figure 17:**
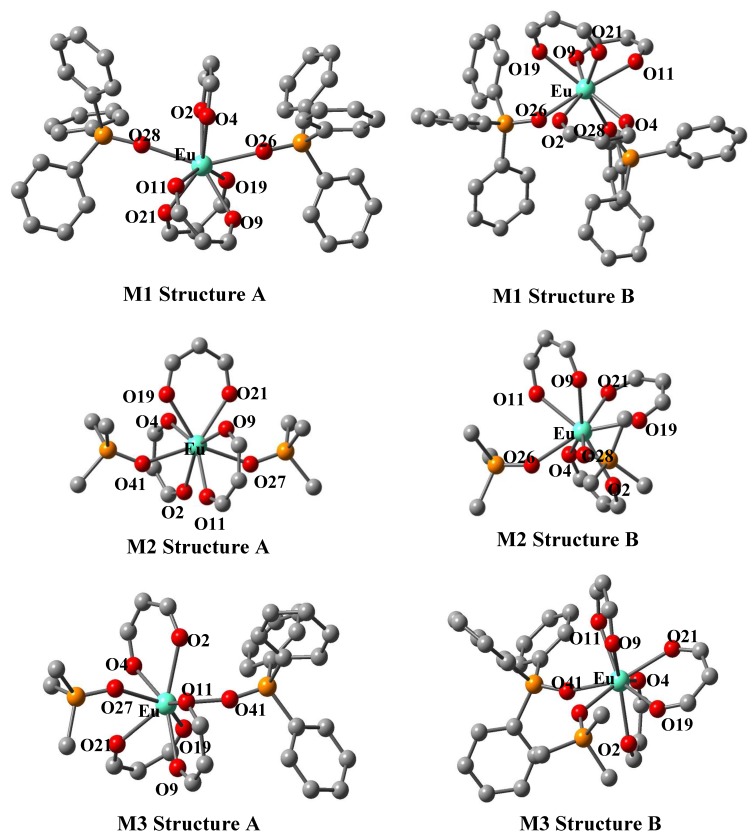
Two optimized geometries for Eu(III) complexes (structures A and B) in the molecular structure shown in Figure 1, M1: R^1^=R^2^=Ph, R^3^=R^4^=H; M2: R^1^=R^2^=Me, R^3^=R^4^=H; M3: R^1^=Me, R^2^=Ph, R^3^=R^4^=H.

Eight-fold coordinated Eu(III) complexes were reported to have a square-antiprism structure [32]. Two coordination geometries (structures A and B) are considered (Figure 17). For structure A, each oxygen atom of a phosphine oxide coordinates in another square in the square-antiprism structure; for structure B, each oxygen atom of phosphine oxides coordinates at the adjacent point of a square in the square-antiprism structure. Structure A was determined by Hasegawa *et al.* and Structure B was determined by Batista *et al.* with single-crystal X-ray diffraction [3,6]. 

Density functional theory (DFT) calculation was carried out to theoretically investigate the coordination structures of Eu(III) complexes with three β-diketonates and phosphine oxides. Two minimum energy points corresponding to two different optimized geometries (structures A and B) have been found, and the difference between two minimum energy points is small (less than 1 kcal/mol). From these results, it is known that structures A and B can both exist in a solution or a polymer [33]. Time-dependent density functional theory (TDDFT) calculations suggest that the absorption coefficient of structure A is larger than that of structure B, resulting in a larger emission intensity.

**Figure 18:**
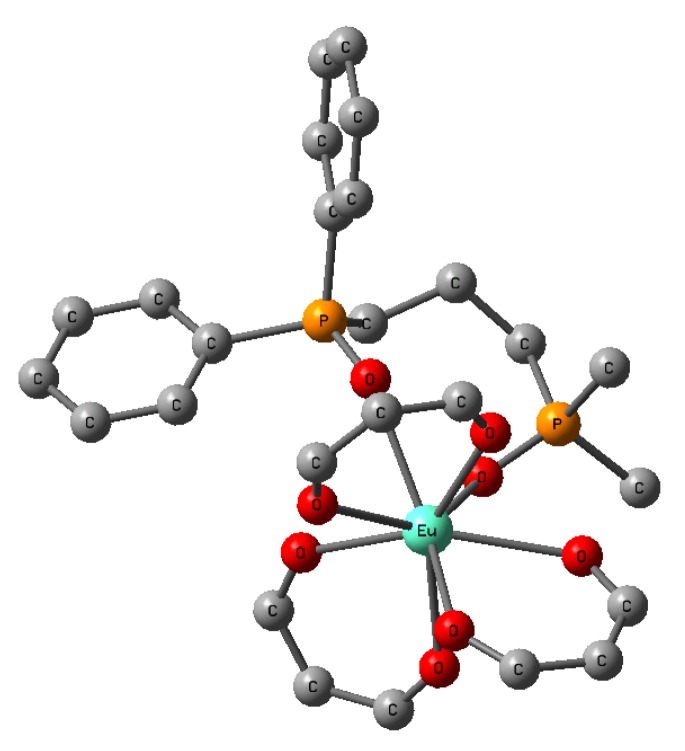
The optimized geometry of simplified Eu(III) complexes with three β-diketonates and non-symmetric phosphine oxides (H atoms are excluded).

A model molecular structure for Eu(III) complexes with three β-diketonates and non-symmetric bis-phosphine oxides (Eu(III) complex **13** was simplified: *t*-Bu groups and C_3_F_7_ groups in β-diketonates were replaced by H atoms and the *n*-Bu groups in the bis-phosphine oxides were replaced by methyl groups) obtained by DFT calculation is shown in Figure 18 [34].

The calculated coordination energy and asymmetry of the ligands field in Eu(III) complexes that have non-symmetric bis-phosphine oxides are larger than those of Eu(III) complexes that have two different phosphine oxides. These results indicate that Eu(III) complexes with non-symmetric bis-phosphine oxides are more stable and have larger emission intensities. A comparison of spectra intensities for Eu(III) complexes with non-symmetric bis-phosphine oxide with that of with two different phosphine oxides is shown in Table 6. The intensity of former complexes is much larger than that of the latter. Moreover, the half width of the former complex is larger than that of latter, thus indicating that the coordination fields of the former complex is more asymmetrical than that of latter. These experimental results correspond well with the calculated results. The λ_max_ in each solution are in the order (mixture of **1** (Figure 3) and non-symmetric bisphosphine oxide **11)**, (**1**, TOPO, and TPPO), (**1)**. Basically, the parity-forbidden f-f electronic transition of Eu(III) ion shifts little with ligands and solvent. The wavelength shifts shown in Table 6 are due to the differences between steric coordination structures.

Sixteen isomers can be considered in Eu(III) complexes with three β-diketonates and one non-symmetric bis-phosphine oxide, but the ratio of existence for the most stable isomer (shown in Figure 18) is found to be about 51%. The sum of the ratios of existence for the six most stable isomers is about 100%, assuming the Boltzmann distribution (*T* = 300 K). The coordination structures of the six most stable isomers in the ground states are similar, and asymmetric ligands fields can be expected.

**Table 6:** Comparison of the spectra intensities of Eu(III) complexes containing non-symmetric bis-phosphine oxide with that of Eu(II) containing two different phosphine oxides (measured in 2,3-dihydrodecafluoropentane at 2 × 10^−4^ M).

Eu(III) complex	Emission intensity (arb. unit)	_λ max_
**1**	209.7	615
**1**+TOPO(1 equation)+TPPO(1 equation)	1159	616
**1**+**11**(1 equation)(non-symmetric bis-phosphine oxide)	1602	618

### 2.11. Development of colorless and transparent emission materials and their applications

**Figure 19:**
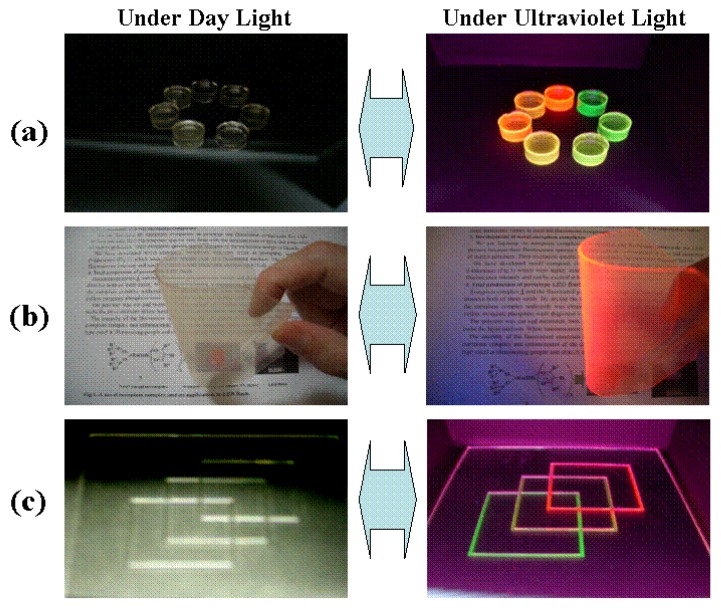
Colorless and transparent emission materials produced by dissolving Eu(III) complexes and/or Tb(III) complexes in a polymer. (a) block, (b) flexible sheet, (c) print on glass.

Eu(III) complexes or Tb(III) complexes, or both, were dissolved in a polymer, and colorless and transparent emission materials were produced (Figure 19) [35,36]. Under daylight, polymers, including rare-earth complexes, are colorless and transparent. On the other hand, under ultraviolet light and near-ultraviolet light, they emit red, green, yellow, and orange colors. Eu(III) complexes that have naphthyl groups in β-diketonates can radiate pure red color when irradiated by not only ultraviolet and near-ultraviolet light, but also by blue light.

**Figure 20:**
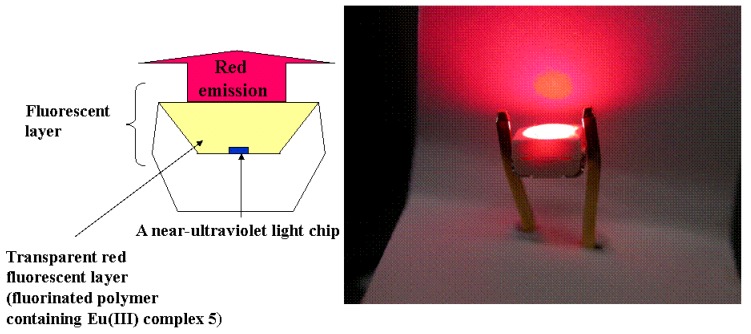
LED devices containing novel Eu(III) complexes in the fluorescent layer.

Ultraviolet-light-excited LED devices (emit red light) with a fluorescent layer consisting of fluorinated polymer and Eu(III) complexes with two different phosphine oxides are shown in Figure 20 [23].

The largest luminous flux obtained under optimum conditions, which to our knowledge is the best result reported so far, was 870 m lumen / 20 mA, when excited by an LED chip whose maximum emission wavelength was 402 nm [23]. 

**Figure 21:**
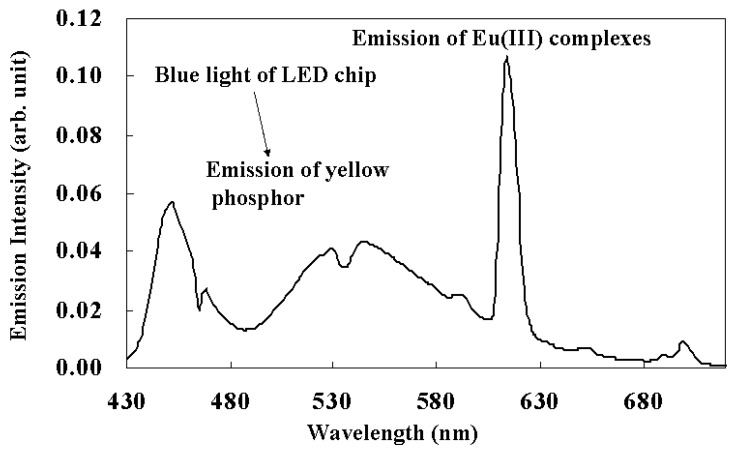
The emission spectra of white LED devices using Eu(III) complexes with two different phosphine oxides.

Eu(III) complexes were applied in the fluorescence layer of white light-emitting diode (LED) devices as red emission compounds. The emission spectra of white LEDs are shown in Figure 21.

The relative spectrum intensity of the red light region is drastically enhanced when compared with previously reported white LEDs. In the case of the emission spectra of previous white LED devices that consist of a blue LED chip and yellow phosphor, the spectra intensities of the red light region are small. Under this LED light, a human looks very pale. On the other hand, our new white LED has an emission spectrum with a large intensity for the red light region because of the effect of emission of Eu(III) complexes. Thus, a human looks much more vividly under the new LED [37,38,39,40,41]. A wide range of color temperatures can be realized by adjusting the strength of red emission.

**Figure 22:**
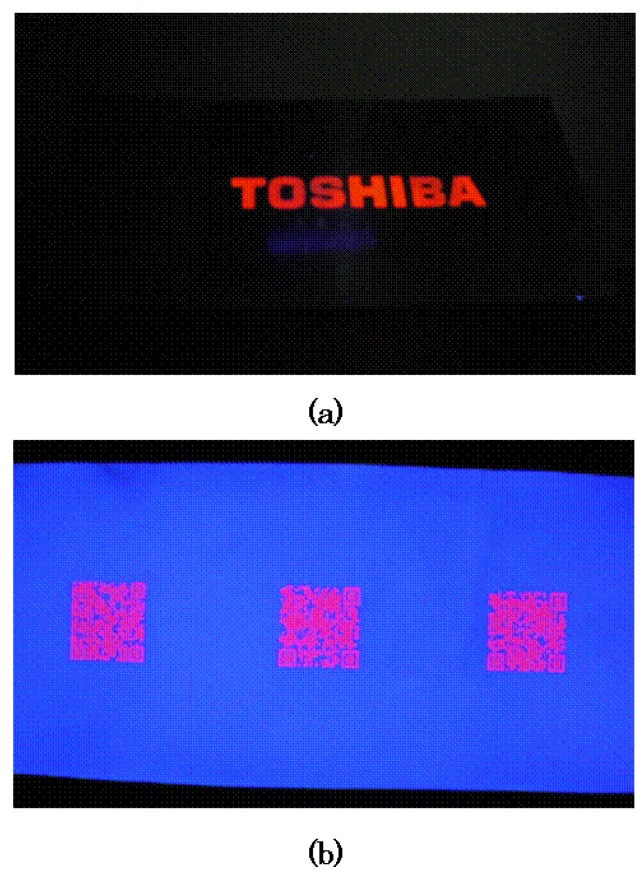
(a) and (b) show a stainless steel sheet and a paper printed via ink jet systems with transparent ink that contains the novel Eu(III) complex. The printed transparent material is invisible under daylight and appears clearly under ultraviolet light. Many applications are expected because any pattern of images can be created on various materials by ink jet systems.

## 3. Conclusion

Novel rare-earth complexes with two different phosphine oxide structures were developed. They are highly soluble even in fluorinated solvents and polymers, and have large emission intensities. They are considered to be eminently suitable for use in LED devices and many other emission devices. The increase of both solubility and emission intensity expands their fields of application and improves the performances of devices. The melting points and thermodynamic properties of Eu(III) complexes are correlated with solubility and NMR analysis explains the correlations between their coordination structures and emission intensity. Ultraviolet-light-excited light-emitting diodes (LED), using our Eu(III) complexes as red luminescence materials, have very high performances (20 mA, 870 m lumen, red). It was also found that a human looks much more vividly under the white LEDs consisting of blue LED chips, inorganic yellow phosphors, and Eu(III) complexes due to the effects of the much larger spectra intensity of the red light region. On the other hand, colorless and transparent emission media, including novel rare-earth complexes, have excellent transparency and strong pure color emission. Any shapes and patterns can be formed by using the ink jet system and are useful for security systems.
